# Navigating the Complexities of Cancer Treatment-Induced Hypertension

**DOI:** 10.3390/jcdd12060235

**Published:** 2025-06-19

**Authors:** Jose Arriola-Montenegro, John Roth, Maria L. Gonzalez Suarez

**Affiliations:** 1 Division of Nephrology and Hypertension, Mayo Clinic, Rochester, MN 55905, USA; arriolamontenegro.jose@mayo.edu; 2 Department of Medicine, Mayo Clinic, Rochester, MN 55905, USA; roth.john@mayo.edu

**Keywords:** cancer, hypertension, onconephrology, VEGF

## Abstract

Cancer therapy-induced hypertension (HTN) is an increasingly recognized complication associated with a wide range of anticancer agents, including vascular endothelial growth factor (VEGF) inhibitors, proteasome inhibitors, tyrosine kinase inhibitors, and alkylating agents. The pathogenesis of HTN in this setting is multifactorial, involving mechanisms such as endothelial dysfunction, nitric oxide (NO) suppression, sympathetic nervous system activation, and vascular remodeling. Additional factors, including paraneoplastic syndromes, poorly controlled pain, mood disturbances, and overlapping cardiovascular risk factors like obesity and diabetes, further contribute to the complexity of diagnosis and management. Despite its prevalence and clinical implications, cancer therapy-induced HTN is often addressed using general population guidelines, with limited oncology-specific protocols available. Accurate blood pressure measurement and individualized treatment plans are critical to optimize outcomes and avoid interruptions to cancer therapy. Antihypertensive agents such as angiotensin-converting enzyme (ACE) inhibitors, angiotensin receptor blockers (ARB), and calcium channel blockers have shown efficacy in both blood pressure control and, in some cases, oncologic outcomes. A multidisciplinary approach involving oncologists, cardiologists, and primary care providers is essential to navigate the interplay between cancer treatment and cardiovascular health. Ongoing research is needed to develop targeted guidelines and improve the long-term care of cancer patients affected by treatment-induced HTN.

## 1. Introduction

In recent decades, the number and diversity of cancer treatments have significantly increased, largely due to the development of targeted pharmacologic interventions [1]. While this expansion has greatly improved the prognosis for many cancer types, it has also revealed previously underrecognized toxicities, particularly those affecting the cardiovascular system [2]. Cancer therapy-induced hypertension (HTN) has emerged as a prominent and increasingly recognized cardiovascular complication. This includes various antineoplastic drug classes such as vascular endothelial growth factor (VEGF) inhibitors, proteasome inhibitors, and alkylating inhibitors, among others [2,3,4]. The International Cardio-Oncology Society (IC-OS), American Heart Association (AHA), and other professional organizations have recognized the urgent need to address cancer therapy-induced HTN [5,6]. As the use of these advanced treatments grows, understanding the relationship between HTN and cancer becomes more critical.

One complicating factor in cancer therapy-induced HTN is the frequent co-occurrence of acute kidney injury (AKI). The kidneys play a central role in blood pressure regulation through mechanisms like the renin–angiotensin–aldosterone system (RAAS), pressure natriuresis, and their interaction with the sympathetic nervous system [7,8,9,10]. Cancer patients are particularly vulnerable to renal damage, whether due to tumor invasion, treatment-related nephrotoxicity, hematopoietic stem cell transplant (HSCT) engraftment syndromes, tumor lysis syndrome (TLS), viral nephropathies, or common malignancy-related issues like sepsis and volume depletion [11,12,13,14]. Recurrent renal damage in this context can disrupt blood pressure (BP) regulation, further exacerbating HTN and adding complexity to the clinical picture [2].

In addition to the direct effects of cancer therapies, other malignancy-related factors further contribute to the development of HTN. These include poorly controlled pain, mood disorders such as anxiety and depression, and sleep disturbances, which are common amongst cancer patients [15,16,17,18]. Additional shared risk factors such as smoking, obesity, and diabetes further compound the risk [3,5,19]. Paraneoplastic HTN also represents another contributor to secondary HTN [20,21,22,23]. These diverse and interacting factors further complicate diagnosis and management of this condition.

Despite growing recognition, there remains a relative lack of dedicated studies and evidence-based guidelines to address this clinical entity. In practice, HTN in cancer patients is often treated empirically using standard guidelines for the general population [6]. However, the unique context of cancer and its treatments requires a more detailed approach. This review aims to highlight the pathophysiology of cancer therapy-induced HTN across various treatment classes, as well as current guidelines regarding its diagnostic approach and management of this unique clinical condition.

## 2. Paraneoplastic Hypertension

Paraneoplastic syndrome refers to cancer-associated signs caused by functional peptides, hormones, or immune cross-reactivity between tumor and normal host tissues [24]. Hypertension is a known paraneoplastic manifestation in various cancers, including renal tumors, neuroendocrine tumors, liver tumors, and, more rarely, ovarian tumors and pelvic teratomas [21,25,26,27,28].

Clear renal cell carcinoma is one of the most common tumors associated with paraneoplastic hypertension, affecting up to 64% of patients [21]. HTN in these cases is driven by the upregulation of the RAAS system, secretion of vasoactive peptides such as endothelin (ET-1), and deficiency of adrenomedullin, which can mediate BP [21,29,30]. Adrenomedullin is a potent vasodilator and natriuretic peptide, and studies have shown that its infusion leads to a reduction in BP [31,32]. Therefore, reduced levels of adrenomedullin may be consistent with the development of hypertension in these patients [33,34].

Pheochromocytomas and neuroendocrine tumors arise from chromaffin cells in the adrenal medulla, leading to unregulated catecholamine secretion. The classic triad of symptoms includes episodic headaches, sweating, and tachycardia, with patients developing sustained or paroxysmal HTN [35]. Hypertension occurs in about 80% of patients with pheochromocytoma, with half developing sustained HTN, 45% presenting with paroxysmal HTN, and 5–15% being normotensive [36,37,38,39]. Hypertension is primarily driven by norepinephrine release, and sustained HTN correlates with this release [40], while paroxysmal HTN is more common in patients with high plasma epinephrine levels [39].

Hepatocellular carcinoma (HCC), the most common form of primary liver cancer [41,42], is also associated with HTN [26,43]. Studies show an increase in systolic blood pressure in HCC patients with concurrent diabetes mellitus [44]. Hypertension is a significant paraneoplastic phenomenon in HCC patients and is considered a potential predictor of survival [43]. The mechanism of HTN in HCC is thought to be caused by abnormalities in the RAAS system [45]. Previous reports have shown significant dysregulation of RAAS, including elevated renin levels, overproduction of angiotensin II, and a subsequent eight-to-tenfold increase in angiotensin II [26,45].

## 3. Etiologies of Cancer Treatment-Induced Hypertension

### 3.1. VEGF Signaling Pathway Inhibitors

VEGF signaling pathway inhibitors (VSPIs) are a widely utilized class of targeted pharmacotherapy in cancer treatment. These medications are clinically indicated for a variety of solid tumors, including colorectal carcinoma, renal cell carcinoma, non-small cell lung cancer, glioblastoma, and female reproductive cancers such as ovarian and cervical cancers [46,47,48,49,50,51,52]. The general, underlying principle is inhibition of angiogenesis primarily via disruption of the VEGF signaling cascade, a key regulator in the formation of new blood vessels [53]. The exact mechanism varies by agent, such as monoclonal antibodies targeting VEGF ligands in the case of bevacizumab as well as those against intracellular VEGF receptor tyrosine kinases, including sunitinib and sorafenib [54]. Collectively, these agents curtail tumor growth, invasion, and metastasis by reducing oxygen and nutrient delivery [55].

The efficacy of VSPIs has been repeatedly demonstrated in clinical trials, as in the case of AViSTAST, which investigated the use of bevacizumab in combination with traditional chemotherapy regimens in metastatic colorectal carcinoma [46]. Patients undergoing VSPI therapy displayed a significant increase in progression-free survival (PFS) and overall survival (OS) compared to standard chemotherapy regimens alone [46]. Likewise, increased PFS was noted with the use of sunitinib and sorafenib in metastatic renal cell carcinoma as compared to interferon-alpha [56,57]. In patients with non-small cell lung cancer, the addition of bevacizumab to carboplatin and paclitaxel resulted in significant improvements in OS as part of the ECOG 4599 trial [49]. Collectively, the results of these studies and similar investigations have repeatedly demonstrated the efficacy of VSPIs in the treatment of malignancy; however, they have also yielded significant insight into adverse events associated with these medications. HTN and proteinuria have notably emerged as frequent and clinically relevant complications associated with VSPI therapy, necessitating careful monitoring and management strategies [58,59].

HTN is a well-known and relatively common side effect in cancer patients treated with VSPIs, with an incidence of approximately 23.0%, although this can vary significantly based on the individual agent and dose [60,61,62]. Predisposing risk factors include patient factors such as female sex, advanced age (>60 years old), elevated BMI (>25 kg/m^2^), pre-existing HTN, and baseline renal dysfunction [63]. More recently, studies have correlated the development of HTN with low circulating plasma levels of VEGF-A and angiopoietin-2, as well as genetic variants of KCNAB1, which is involved in the production of voltage-gated potassium channels [64].

The pathophysiology of VSPI-induced HTN is complex and largely attributed to reduced nitric oxide (NO) production and vascular endothelial dysfunction [65]. VEGF-induced NO production promotes vasodilation, and disruption of this signaling cascade raises systemic vascular resistance, contributing to elevations in blood pressure [65]. Beyond systemic vasoconstriction, VSPIs are believed to influence renal sodium handling and glomerular filtration, further contributing to the development of HTN [65]. Consequently, patients who develop VSPI-induced HTN are at increased risk for a variety of complications. This includes stroke, myocardial infarction, and renal injury, all of which carry downstream effects on cancer treatment through dose reductions or even drug discontinuation [66].

Proteinuria is another well-characterized toxicity associated with VSPI therapy. Incidence has been estimated at 18.7%, although this can vary depending on the specific agent [67]. Patient-related risk factors include Asian ethnicity, diabetes, elevated baseline blood pressure, and prior nephrectomy, while medication-related factors include specific VSPI agents, increased dose, and treatment duration [68,69]. The underlying mechanism for VSPI-induced proteinuria involves damage to the glomerular endothelium and podocyte dysfunction. VEGF signaling is a key regulator for glomerular endothelial integrity, and disruption contributes to foot process effacement, which ultimately increases glomerular permeability [59,70]. In severe cases, VSPI-induced proteinuria may progress to nephrotic syndrome [71].

### 3.2. Proteasome Inhibitors

Proteasome inhibitors, such as bortezomib, carfilozib, and ixazomib, are commonly used in the treatment of multiple myeloma, primarily targeting the ubiquitin–proteasome system [72,73]. These anticancer drugs have been linked to high blood pressure, which affects approximately 10% of patients, although this varies by agent [74]. This toxicity is driven by multiple mechanisms, including angiotensin-induced HTN, aortic vascular remodeling, dysregulated NO homeostasis, and vasoconstriction [75,76,77]. Additionally, the accumulation of ubiquitinated proteins from proteasome inhibition leads to cellular apoptosis and endothelial damage, which can exacerbate cardiovascular adverse effects [73]. Moreover, numerous studies have demonstrated a strong association between carfilzomib and both new onset and worsening HTN [78,79,80], as well as other cardiac events such as heart failure, arrhythmias, and cardiac arrest [81].

### 3.3. Bruton’s Tyrosine Kinase (BTK) Inhibitors

BTK inhibitors are commonly used in the treatment of chronic lymphocytic leukemia, mantle cell lymphoma, and Waldenstrom macroglobulinemia [82,83,84]. However, these medications are well recognized for their association with the development of HTN [85,86,87]. This complication is attributed to multiple mechanisms, including VEGF inhibition, vascular fibrosis, cellular remodeling due to PI3K pathway inhibition, downregulation of NO, and endothelial dysfunction evidenced by an increase in plasma ET-1 and a decrease in plasma renin [88,89,90]. Studies have shown that the incidence of all-grade HTN is 23.4%, with 5.7% of patients experiencing high-grade HTN [91].

### 3.4. Rapidly Accelerated Fibrosarcoma B-Type (BRAF) and Mitogen-Activated Extracellular Signal-Regulated Kinase (MEK) Inhibitors

BRAF/MEK inhibitors target the Ras-RAF-MEK-ERK pathway and are commonly used for treatment of metastatic melanoma [92]. An important side effect of this dual treatment is the development of HTN, which is caused by the upregulation of CD47. This upregulation inhibits the NO/cyclic guanylyl monophosphate pathway, reducing NO production, which promotes vasoconstriction [92,93,94,95]. The incidence of HTN has been reported to be approximately 20% with BRAF/MEK inhibitors and 14% with BRAF inhibitor monotherapy [96]. Additional side effects due to Ras-RAF-MEK-ERK pathway inhibition include left ventricular systolic dysfunction, atrial arrhythmia, QT interval prolongation, and venous thromboembolism [96,97,98].

### 3.5. Radiation Therapy

Radiation therapy is an antineoplastic treatment that can predispose individuals to HTN through three main mechanisms: disruption of the carotid baroreceptor function, radiation nephropathy, and renal radiation-induced artery stenosis [99]. Specifically, neck radiation can lead to injury of the carotid arteries, causing atherosclerosis and fibrosis, which may reduce distensibility of the carotid sinus [100]. Impaired stretch-induced afferent carotid sinus nerve activity promotes chronic attenuation of both vagal and sympathetic baroreflex sensitivity. This dysfunction predisposes individuals to unrestrained sympathetic activation, which is evidenced as labile blood pressure, HTN, orthostatic intolerance, and tachycardia [101,102].

Radiation nephropathy is categorized in two stages based on the time after radiotherapy [103]. Acute radiation therapy occurs 6–18 months post-treatment. This is characterized by glomerular damage-related complications, such as proteinuria, edema, and azotemia [103,104]. Kidney biopsy findings in the acute phase primarily demonstrate vascular and glomerular changes: occluded capillary loops, loss of endothelial cells with subendothelial expansion, and mesangiolysis [105,106]. In contrast, chronic radiation nephropathy typically develops after 18 months and is associated with signs of chronic kidney disease, such as HTN, albuminuria, small atrophic kidneys, and anemia [103,107]. Common kidney biopsy features in the chronic stage include renal interstitial fibrosis, loss of nephron mass, and sclerosis of interlobular and arcuate arteries [106].

Renal radiation-induced artery stenosis is uncommon and typically affects the proximal segment of the renal artery. It typically develops after prolonged infradiaphragmatic radiation therapy and often presents as new-onset resistant HTN and elevated serum creatinine [108,109].

### 3.6. Alkylating Agents

Alkylating agents, such as cyclophosphamide, ifosfamide, busulfan, and cisplatin, are used in the treatment of hematologic malignancies (lymphoma and leukemia) and solid organ malignancies (testicular, brain, and ovarian cancers) [110,111]. These antineoplastic agents are associated with HTN via different mechanisms, including oxidative stress, cellular toxicity, endothelial damage, reduced VEGF concentrations, decreased NO bioavailability, direct vascular toxicity, and nephrotoxicity [75,112,113,114,115]. Cisplatin-based chemotherapy has been associated with an increased incidence of HTN as a late side effect for testicular cancer survivors [116]. Additionally, elevated blood pressure was common in cancer survivors after receiving treatment with ifosfamide [117]. Hypertension was evidenced in 25% to 36% of adults who received busulfan and in 58% of pediatric patients [118].

### 3.7. Platinum-Based Compounds

Platinum-based chemotherapies exert their neoplastic effects by inducing DNA cross-linking, thus inhibiting replication and promoting apoptosis [119]. These drugs are associated with the development of cancer treatment-induced HTN. This is thought to be multifactorial and primarily mediated through their direct effects upon the vasculature [119]. Vascular endothelial cells are vulnerable to the apoptotic effects of platinum derivatives, promoting cell death and a generalized inflammatory state [114]. Furthermore, platinum-containing drugs also promote endothelial dysfunction through a variety of mechanisms, including decreased production of NO [120]. There is growing evidence additionally linking the chronic neurotoxic effects of platinum agents to sympathetic dysfunction, further contributing to elevated blood pressure through downstream central nervous system-mediated reflexes [121]. Collectively, these effects promote increased systemic vascular resistance through widespread vasoconstriction, which induces the development of HTN [114,119,120,121,122]. Importantly, the hypertensive effects of platinum-based therapies may become chronic, as investigations have shown evidence of persistently elevated blood pressures even decades after exposure [122].

### 3.8. BCR-ABL Tyrosine Kinase Inhibitors

BCR-ABL tyrosine kinase inhibitors (TKIs) are selective, monoclonal antibodies that prevent cell division and ultimately promote apoptosis through downstream effects [123]. They are primarily utilized in the treatment of chronic myeloid leukemia and Philadelphia chromosome-positive acute lymphoblastic leukemia. These agents have been linked to various cardiovascular toxicities, which may be attributable to off-target inhibition of non-BCR-ABL TKIs [123]. However, the exact mechanisms behind these effects are still being investigated and are not always applicable to the entire class of medications. Up to 20–30% of cancer patients treated with ponatinib develop HTN thought to be attributed to inhibition of the VEGF system [124,125]. Nilotinib is another agent with an expansive cardiovascular risk profile, including the development of HTN. Studies have shown nilotinib to promote atherosclerosis as well as suppress endothelial cell proliferation, both of which likely contribute to the development of high blood pressure [126]. Given the distinct adverse event profiles of different TKIs, more investigation is required to better elucidate how other agents may contribute to the development of HTN [124].

### 3.9. Mammalian Target of Rapamycin Inhibitors

Mammalian target of rapamycin (mTOR) inhibitors, such as everolimus and sirolimus, are used as third-line treatment for renal cell carcinoma [56,127] and ovarian cancer [128]. In vitro studies have shown that mTOR inhibition decreases VEGF secretion, which may predispose patients to HTN [129,130]. Additionally, research suggests that mTOR inhibitors may induce HTN through increased oxidative stress and sympathetic activation, leading to afferent arteriolar vasoconstriction [131]. Everolimus has been associated with the development of all-grade HTN in patients with metastatic renal cell carcinoma [130]. Furthermore, the combination of everolimus and lenvatinib, a VEGF inhibitor, was associated with all-grade HTN in 41% of patients and high-grade HTN in 14% of patients [130]. Moreover, mTOR inhibitors impair glucose and lipid metabolism and contribute to abdominal obesity, which may further increase the cardiovascular risk associated with these agents [132,133].

### 3.10. Anti-Androgen Therapy

Anti-androgen therapy is a cornerstone of prostate cancer treatment and includes multiple classes of medications, such as androgen receptor antagonists such as cyproterone acetate, flutamide, enzalutamide, and bicalutamide, as well as 17α-hydroxylase/C17,20-lyase (CYP17) inhibitors that block androgen synthesis, such as abiraterone [134]. These agents have well-established metabolic and cardiovascular risk profiles, including the development of HTN. Abiraterone inhibits production of both androgens and cortisol through its effect upon CYP17 enzymes [134]. With the loss of the cortisol-mediated negative feedback loop, subsequent overexpression of adrenocorticotropic hormone results in a buildup of mineralocorticoid precursors and development of secondary HTN [134]. The exact mechanism by which androgen receptor antagonists are associated with elevated blood pressure is less clear. A higher incidence of HTN was noted in those prostate cancer patients treated with enzalutamide [135]. Similarly, in the context of gender-affirming hormonal therapy, cyproterone acetate has been associated with increased blood pressure and HTN onset [136]. More investigation is needed to fully elucidate the cardiovascular effects of these agents.

### 3.11. Adjuvant Therapies

Many cancer patients receive adjuvant therapies alongside chemotherapy as part of their treatment plan. These therapies may contribute to the development of HTN or exacerbate pre-existing HTN [118,137,138]. Medications such as erythropoiesis-stimulating agents (ESAs), glucocorticoids, nonsteroidal anti-inflammatory drugs (NSAIDs), and calcineurin inhibitors are commonly involved and are known to cause HTN [137,138]. Therefore, it is essential to carefully monitor patients when these drugs are included in the cancer treatment regimen, especially when used in combination with antineoplastic agents known to increase blood pressure.

ESAs have been associated with an increased risk for HTN in multiple clinical trials [137]. Various mechanisms have been identified, including increased blood viscosity, arterial remodeling, and increased vascular resistance to the vasodilator effects of NO, which predisposes patients to vasoconstriction [139,140].

Glucocorticoids are known for their side effects, including water and sodium retention, which can lead to a rise in blood pressure [141]. Glucocorticoid-induced HTN has been reported in up to 13% of patients [142].

NSAIDs are recognized for their significant association with HTN [143]. These side effects are primarily driven by their inhibitory effect on the production of vasodilatory prostaglandins and retention of water and salt [144]. Studies have shown an increase in supine mean blood pressure by 5.0 mmHg [143].

Through the inhibition of T-cell function, calcineurin inhibitors prevent the rejection of transplanted solid organs and graft-versus-host disease in the context of allogeneic bone marrow transplantation. These medications have been shown to be associated with HTN. Mechanisms include activation of RAAS and the sympathetic nervous system, increased proximal tubule sodium reabsorption, endothelial dysfunction via stimulation of endothelin-1, inhibition of NO production, and increased oxidative stress [145,146]. Additionally, these medications may predispose patients to nephrotoxicity, which can contribute to prohypertensive effects [145]. Some 30–80% of patients treated with calcineurin inhibitors develop HTN [147,148].

**Figure 1:**
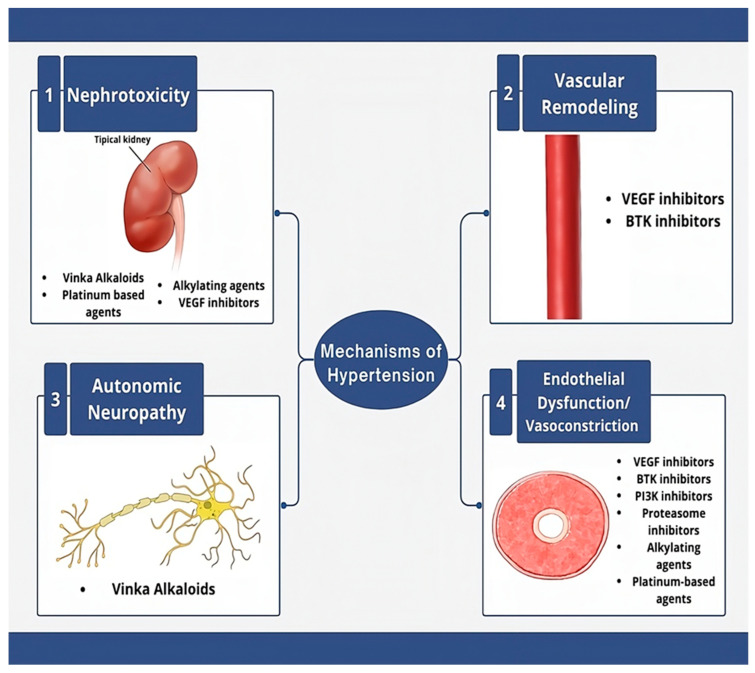
Mechanisms of hypertension.

A graphical summary of the key mechanisms involved in cancer treatment-induced hypertension is provided in Figure 1. Additionally, Table 1 outlines the mechanisms, representative medications, and relative incidence of all-grade hypertension across major oncologic drug classes, while Table 2 highlights the hypertensive impact of radiation and adjuvant therapies. Together, these tools aim to support risk stratification and clinical decision-making in this complex population.

## 4. Diagnosis of Hypertension in Cancer Patients

Accurate measurement of BP is crucial for diagnosing and managing HTN, particularly in cancer patients. These patients may experience pain or anxiety during clinic visits or be taking medications such as NSAIDs, ESAs, and corticosteroids, which can interfere with BP readings [6,118].

For accurate measurement, patients should be seated quietly and at rest for 3 to 5 min before their BP is taken. The measurement should be carried out in a quiet room with the patient’s legs resting on the floor (uncrossed) and their back properly supported. The arm used for BP measurement should be at heart level, with a correctly sized, calibrated cuff placed on a bare arm. Additionally, the patient should have an empty bladder and avoid caffeine or smoking for at least 30 min before the measurement [6,118,158,159].

During the clinic visit, at least three BP readings should be obtained and averaged. If any readings are elevated, they should be verified on at least one additional measurement before diagnosing HTN [158]. Automated office BP measurement can be a useful tool for obtaining multiple readings in a single visit and can be performed with or without the presence of a provider [160]. For initial BP readings ≥ 120/70 mmHg, 24-hour ambulatory BP monitoring is recommended to confirm the diagnosis [160]. However, this method is not always feasible to perform more than twice a year for elevated readings [161]. As an alternative, home BP monitoring requires patients to measure BP twice a day for at least five consecutive days. This method is often preferred by both patients and clinicians, as it allows for close monitoring of BP, facilitating medication adjustments and helping identify white coat hypertension [162].

The 2017 American College of Cardiology/American Heart Association (ACC/AHA) Clinical Practice Guidelines and the 2018 ESC/European Society of Hypertension Guidelines are commonly used to diagnose and grade HTN in cancer patients. Hypertension is diagnosed when office-based or average home BP measurements are ≥130/80 mmHg or when the average BP on ambulatory blood pressure monitoring (ABPM) is ≥125/75 mmHg [158,163,164].

It is essential to measure baseline BP before starting prohypertensive anticancer therapies, as some patients may experience a significant increase in BP during treatment, requiring prompt initiation or adjustment of antihypertensive therapy. The National Cancer Institute Investigational Drug Steering Committee recommends targeting a BP of <140/90 mmHg before starting VSPI therapy [165].

## 5. Blood Pressure Goal in Patients with Cancer

Patients with an estimated 10-year atherosclerotic cardiovascular disease (ASCVD) risk of ≥10% or those with additional cardiovascular comorbidities such as type 2 diabetes mellitus, chronic kidney disease, stroke, or peripheral vascular disease should aim for a BP goal of <130/80 mmHg. Patients with an estimated 10-year ASCVD risk of ≤10% and without additional cardiovascular comorbidities should aim for a BP goal of <140/90 mmHg [158].

For asymptomatic patients with metastatic cancer and an expected survival of 1–3 years, the target BP may be increased to 140–159/90–99 mmHg. However, if BP rises above >160/100 mmHg, treatment should be initiated in all patients to prevent life-threatening complications [5,99]. Additionally, prohypertensive anticancer agents should be withheld if BP rises above 180/110 mmHg and should not be restarted until BP is controlled to <160/110 mmHg [4,5]. If a hypertensive emergency occurs, consideration of permanent discontinuation of prohypertensive anticancer agents should be made [99].

## 6. Management of Cancer Therapy-Related HTN

### 6.1. Lifestyle Modifications

Patients should be counseled on lifestyle changes that can help improve BP control. This includes limiting sodium intake, maintaining a healthy weight, reducing caffeine and alcohol consumption, avoiding NSAIDs, and increasing physical activity [6,166,167]. Providers should also address the impact of concomitant therapies that may elevate BP during anticancer therapy, such as corticosteroids or ESAs [168]. Additionally, managing pain, minimizing anxiety, and evaluating and treating comorbidities (e.g., obstructive sleep apnea or diabetes mellitus) that may contribute to high BP are essential steps in effective HTN management [6,44,99].

### 6.2. Antihypertensive Medications

Currently, there are no specific antihypertensive guidelines for cancer patients undergoing anticancer therapies, and antihypertensive management should follow the general population’s guidelines [169]. However, further recommendations emphasize that HTN management should be a multidisciplinary approach, including cardiologists, oncologists, nurses, primary care physicians, patients, and their families [6].

The choice of antihypertensive agent should be individualized based on the type of anticancer treatment. For instance, non-dihydropyridine calcium channel blockers (e.g., diltiazem and verapamil) should be avoided in patients receiving therapies metabolized by P-glycoprotein and cytochrome P450 3A4 [170] as well as sunitinib and sorafenib due to known interactions [171]. First-line treatments typically include angiotensin-converting enzyme (ACE) inhibitors, angiotensin receptor blockers (ARBs), dihydropyridine calcium channel blockers (CCB), or diuretics [172,173,174,175,176].

ACE inhibitors and ARBs are considered the first-line treatment for anti-VEGF-induced HTN [177]. This is due to their renoprotective effects, which are particularly beneficial for diabetic patients at risk of proteinuria during VEGF inhibition therapy [178]. These agents are particularly effective in managing mild increases in BP (10–15 mmHg) during initiation of anticancer treatment [178]. Experimental studies have shown that losartan and captopril reduce VEGF expression in renal tumors, suppress tumor size, and shrink lung metastases in renal cell carcinoma patients [179]. Furthermore, ACE inhibitors and ARBs have been associated with improved survival in metastatic renal cell carcinoma patients [180] and lower colorectal cancer risk following a negative baseline colonoscopy [181].

Dihydropyridine CCBs such as amlodipine have been associated with improvements in de novo HTN and worsening HTN in patients undergoing treatment with bevacizumab for advanced or metastatic non-small cell lung carcinoma, colorectal cancer, or ovarian cancer [182]. Additionally, CCBs and potassium-sparing agents were associated with the largest reductions in BP in patients on anti-VEGF tyrosine kinase inhibitor therapy for metastatic renal cell cancer [174]. Thiazide diuretics are an acceptable alternative. However, caution must be considered in patients at risk of volume depletion secondary to chemotherapy [6].

Sodium–glucose cotransporter 2 (SGLT2) inhibitors are emerging as a potential adjunctive therapy for cancer therapy-induced HTN. Beyond their glycemic benefits, SGLT2 inhibitors have demonstrated modest blood pressure-lowering effects and significant cardiorenal protection [183]. SGLT2 inhibitors have been shown to reduce endothelial dysfunction through a variety of mechanisms, which may be particularly advantageous in those treated with VSPIs or other classes known to disrupt endothelial homeostasis [184]. Moreover, empagliflozin has been shown to reduce sunitinib-induced HTN in addition to other treatment-related cardiac toxicities [185]. As cancer patients have often been excluded from randomized controlled trials involving SGLT2 inhibitors, more research is needed to explore the cardiovascular benefits of these medications in cancer patients.

**Figure 2:**
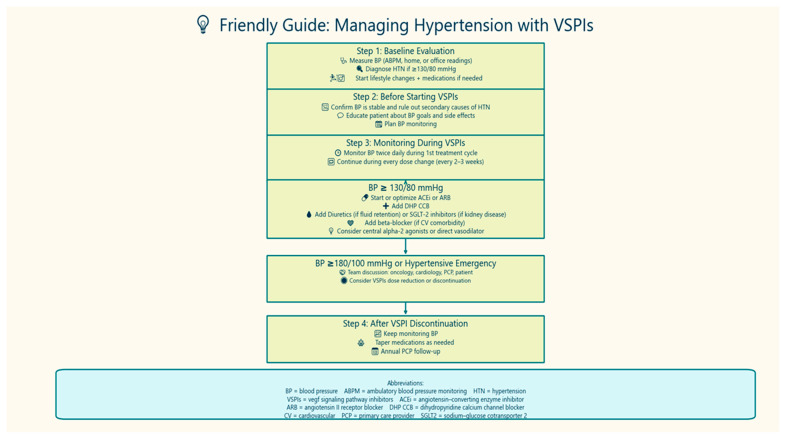
Friendly Guide: Managing Hypertension with VSPIs.

To enhance clinical applicability, we have incorporated a practical flowchart (Figure 2) that outlines a stepwise approach to managing hypertension in patients receiving VSPIs, integrating both pharmacologic and non-pharmacologic interventions. This aims to guide clinicians in tailoring management strategies based on oncologic regimen and patient-specific risk factors. Additionally, we acknowledge critical gaps in the field, including the absence of standardized blood pressure monitoring protocols during cancer therapy, limited comparative studies evaluating antihypertensive strategies in this population, and the lack of cardio-oncology-specific BP targets in current guidelines. These gaps underscore the urgent need for further research to optimize cardiovascular care in oncology patients.

## 7. Conclusions

Cancer therapy-induced hypertension is a common and clinically significant complication that requires early recognition and individualized management. While current treatment often follows general hypertension guidelines, the unique risks associated with cancer therapies necessitate a tailored, multidisciplinary approach. Further research is essential to develop dedicated guidelines and improve outcomes for cancer patients affected by treatment-related hypertension.

## Figures and Tables

**Table 1:** Classes of Antineoplastic Agents Associated with Hypertension: Mechanisms of Action, Representative Medications, Relative Incidence of All-Grade Hypertension, and Proposed Pathophysiological Mechanisms.

Medication Class	Mechanism of Action	Example Medications	Relative Incidence of All-Grade HTN †	Impaired NO Homeostasis	Vascular Endothelial Dysfunction	Capillary Rarefaction	Impaired Natriuresis	Angiotensin-Mediated Vasoconstriction	Direct Vascular Toxicity	Abnormal Vascular Remodeling	Oxidative Stress	Impaired VEGF Signaling	Renal Toxicity	Sympathetic Dysfunction
VSPI	Disruption of VEGF-mediated angiogenesis	Ramucirumab [149] (anti-VEGF-R2 Ab )	↑	+	+	+	+					+		
		Bevacizumab [150] (anti-VEGF-A Ab)	↑											
		Aflibercept [151] (anti-VEGF-Trap ligand Ab)	↑↑											
		Pazopanib [152] (tyrosine kinase inhibitor)	↑↑											
Proteosome inhibitors [6,73,75,76,77]	Inhibition of ubiquitin-proteosome cascade	Carfilzomib	↑↑	+	+			+		+				
		Bortezomib	↑											
Alkylating agents [6,75,112,113,114,115]	Impaired genome replication and transcription via DNA cross-linking	Busulfan	↑↑	+	+				+	+	+	+	+	
		Ifosfamide	↑											
Platinum-containing compounds [6,110,114,119,120,121,122,153]	Impaired genome replication and transcription via DNA cross-linking	Cisplatin	↑↑↑	+	+				+				+	+
		Carboplatin	↑											
mTOR inhibitors [6,129,130,131,154]	Disruption of cellular metabolism, growth, and proliferation via serine-threonine kinase inhibition	Everolimus	↑								+	+		+
		Temsirolimus	↑											
BRAF/MEK [92,93,94,95,96,155]	MAPK pathway inhibition	Encorafenib/binimetinib	↑	+										
		Vemurafenib/cobimetinib	↑											
BTK inhibitors [88,89,90,156]	Impeded B cell receptor signaling	Acalabrutinib	↑	+	+					+		+		
		Ibrutinib	↑↑↑											
BCR-ABL TK inhibitors ‡ [123,124,125,126,157]	Inhibition of BCR-ABL TK-mediated effects upon cellular division and apoptosis	Ponatinib	↑↑		+							+		
		Nilotinib	↑											

VSPI = VEGF signaling pathway inhibitor; VEGF = vascular endothelial growth factor; Ab = antibody; HTN = hypertension; NO = nitric oxide; DNA = deoxyribonucleic acid; mTOR = mammalian target of rapamycin; BRAF = rapidly accelerated fibrosarcoma B-type; MEK = mitogen-activated extracellular signal-regulated kinase; MAPK = mitogen-activated protein kinase; BTK = Bruton’s tyrosine kinase; BCR-ALB = breakpoint cluster region–Abelson leukemia virus 1; TK = tyrosine kinase. † Relative incidence: ↑: <25%; ↑↑: 25–50%, ↑↑↑: 50–75%. + symbol indicates the involvement or contribution of a particular mechanism in the development of hypertension. ‡ mechanisms of BCR-ABL TK inhibitor-induced HTN is not fully characterized. + symbol determines possible mechanism of hypertension.

**Table 2:** Adjuvant and radiation therapies.

Medication Class		Mechanism of Action	Example Medications	Mechanism of HTN
**Adjuvant Therapies**	Corticosteroids	Predominantly glucocorticoid activity	Prednisone, dexamethasone	Predominantly mineralocorticoid-induced water and Na retention
	Calcineurin inhibitors	Impaired T cell activation via inhibition of transcription factors	Cyclosporine, tacrolimus	RAAS activation, sympathetic dysfunction, increased proximal tubule Na resorption (NCC-mediated), endothelial dysfunction (ET1 mediated), oxidative stress, impaired NO homeostasis
	Erythropoiesis stimulating agents	Increased erythrocyte production	Epoetin alfa, epoetin beta	Increased blood viscosity, arterial remodeling, impaired NO homeostasis
	NSAIDs	Decreased prostaglandin synthesis via inhibition of COX enzymes	Ketorolac, ibuprofen	Impaired natriuresis
**Radiation Therapy**		Cellular apoptosis	N/A	Carotid baroreceptor dysfunction, radiation nephropathy, radiation-induced renal artery stenosis

HTN = hypertension; RAAS = renin–angiotensin–aldosterone system; NCC = sodium chloride cotransporter; ET1 = endothelin-1; NO = nitric oxide; NSAIDs = non-steroid anti-inflammatory drugs; COX = cyclooxygenase.

## Data Availability

No new data was generated for this review.
